# Salvage external beam radiotherapy after HIFU failure in localized prostate cancer: A single institution experience

**DOI:** 10.3389/fonc.2022.1028858

**Published:** 2022-11-03

**Authors:** Vanessa Di Lalla, Sara Elakshar, Maurice Anidjar, Marwan Tolba, Toufic Hassan, Boris Bahoric, Victor McPherson, Stephan Probst, Tamim Niazi

**Affiliations:** ^1^ Department of Radiation Oncology, Jewish General Hospital, McGill University, Montreal, QC, Canada; ^2^ Department of Clinical Oncology, Tanta University, Tanta, Egypt; ^3^ Department of Urology, Jewish General Hospital, McGill University, Montreal, QC, Canada; ^4^ Department of Nuclear Medicine, Jewish General Hospital, McGill University, Montreal, QC, Canada

**Keywords:** localized prostate cancer, prostate cancer treatment, salvage radiotherapy, high-intensity focused ultrasound, outcomes

## Abstract

**Purpose/objectives:**

High-intensity focused ultrasound (HIFU) remains investigational as primary treatment for localized prostate cancer but is sometimes offered to select patients. At HIFU failure, data guiding salvage treatment is limited to small retrospective series with short follow-up. We evaluated our institutional experience using salvage radiation therapy (SRT) after HIFU failure.

**Materials/methods:**

We conducted a retrospective analysis of patients with local failure post-HIFU who received salvage image-guided external beam radiation therapy (EBRT) delivered *via* intensity-modulated radiotherapy (IMRT). Our primary endpoint was biochemical failure-free survival (bFFS) defined as prostate-specific antigen (PSA) nadir + 2 ng/mL. Secondary endpoints included metastasis-free survival (MFS) and overall survival (OS). Endpoints were evaluated using Kaplan-Meier analysis.

**Results:**

From 2013 to 2018, 12 out of 96 patients treated with primary HIFU received SRT *via* conventional or moderate hypofractionation. Median time from HIFU to SRT was 13.5 months. Seven patients had stage migration to high-risk disease at the time of SRT. Mean PSA prior to SRT was 8.2ug/L and mean nadir post-SRT was 1.2ug/L. Acute International Prostate Symptom Score (IPSS) as well as International Index of Erectile Dysfunction (IIEF) scores were similar to baseline (p = 0.5 and 0.1, respectively). Late toxicities were comparable to those reported after primary EBRT for localized prostate cancer. At a median follow-up of 46 months, the OS was 100%. The 5-year bFFS and MFS were both 83.3%.

**Conclusions:**

To our knowledge, we report one of the largest series on contemporary SRT post HIFU failure. We show that SRT is feasible, effective and carries no additional acute or delayed toxicity.

## Introduction

Standard primary treatment options for localized prostate cancer include active surveillance, radical prostatectomy, and radiation therapy with or without androgen deprivation therapy (ADT) (1–3). Goals of therapy include effective oncologic control while minimizing long-term side effects. In addition, effective local therapies should reduce risk of metastatic disease progression, which remains a significant burden on healthcare systems and a major cause of cancer-specific mortality (4).

With the aim of decreasing locoregional toxicity, HIFU has been used in men with localized prostate cancer as an alternative to radiation therapy and prostatectomy. Classical indications for HIFU include men aged >65, those who are obese, have contraindications to surgery or refuse surgery (5). Published literature using HIFU in the primary treatment setting has revealed that the procedure is safe, with acceptable oncologic outcomes. Post-procedure positive biopsy rates range from 7-34%, with 5-year biochemical progression-free survival (bPFS) ranging from 30-78% (5–11).

Despite favorable efficacy and safety data, systematic reviews of patients who underwent HIFU as a primary treatment have shown that quality of evidence remains poor given lack of randomized prospective data comparing HIFU to other treatment modalities (12, 13). When retrospectively comparing disease-free survival outcomes with EBRT, HIFU was inferior at 1 year, however this difference did not remain significant with longer follow-up (12). Given the paucity of high-level data to support its use, both European and American urological associations do not recommend HIFU as a primary treatment and still consider it investigational (1–3). Nonetheless, some early localized prostate cancer patients are still offered and treated with HIFU as the primary treatment modality.

For patients who do not achieve a complete response or for those with disease recurrence post-HIFU, options for salvage treatment include radical prostatectomy or EBRT. However, data for salvage therapy post-HIFU remains limited by small, retrospective and short follow up series (14–19).

In the present study, we sought to evaluate our institutional experience in a cohort of patients treated with salvage radiation therapy after primary HIFU failure. Specifically, we aimed to assess oncologic outcomes as well as the feasibility and toxicity profile of modern RT in the salvage setting.

## Materials and methods

### Patient population

We retrospectively reviewed data from all patients who received HIFU at our institution from 2013 to 2018 for biopsy-proven prostate adenocarcinoma. We then collected data on patients who received salvage external beam radiation therapy delivered *via* image-guided intensity modulated radiotherapy (IG-IMRT) at the time of HIFU failure. Persistent disease or local recurrence after HIFU was identified by PSA rise and confirmed by magnetic resonance imaging (MRI) with or without positive tissue biopsy. Bone scan and computed tomography (CT) scan of chest, abdomen and pelvis or functional positron emission technology (PET) imaging were done to rule out distant metastases.

### Radiotherapy procedures

We used a pre-treatment CT scan for the simulation of all patients. Daily specific preparation required patients to have a comfortably full bladder with an empty rectum. Clinical target volumes (CTV), planning target volumes (PTV) and organs at risk (OAR) including the bowel, bladder, rectum, sigmoid and femoral heads were delineated on the pre-treatment CT scan. The CTV included the prostate with the proximal 1cm seminal vesicles. The PTV was generated by expansion of the CTV by a 5-7 mm margin. Pelvic lymph node irradiation was included for all high risk or pelvic lymph node positive patients. For patients with T3b disease, the whole seminal vesicles were included in the CTV.

Treatment was delivered *via* IG-IMRT with either conventional fractionation (76-78Gy in 38-39 fractions, 2Gy per fraction) or hypofractionation (66Gy in 22 fractions, 3Gy per fraction). Simultaneous integrated boost to gross tumor or positive pelvic lymph nodes was also utilized. The planning endpoint was to cover at least 95% of the PTV with the full prescribed dose. Image-guided RT was used in all patients with daily cone-beam CT to assess appropriate bladder filling, rectal emptying and PTV coverage.

During treatment, patients were seen on a weekly basis by their radiation oncologist. At these visits, genitourinary (GU) and gastrointestinal toxicities (GI) were assessed. The Common Terminology Criteria for Adverse Events version 5 (CTCAE v.5) scale was used to report GI toxicity. Acute IPSS as well as IIEF scores were used to report GU and erectile toxicities, respectively. Toxicities were also reported on follow-up visits, with the first follow-up visit occurring within 3 months after completion of RT.

### Data collection and statistical analysis

This study was performed in line with the principles of the Declaration of Helsinki. Approval was granted by the Research Ethics Board of the CIUSSS West-Central Montreal (CIUSSS WCM REB) (2021-2719 (trial number)/Initial approval Feb 01, 2021). Given the retrospective nature of this study, informed consent from participants was not required by the REB.

Patient and disease characteristics were collected at the time of initial diagnosis. Digital rectal examination, PSA blood test and IPSS/IIEF scores were collected immediately post-HIFU and at each follow-up visit. Both acute (>6 months) and delayed (>6 months) toxicity data were collected post-RT, using the CTCAE v.5 scale. Our primary endpoint was bFFS based on the ASTRO Phoenix definition of PSA nadir + 2 ug/L. Secondary endpoints included toxicity associated with SRT, MFS and OS. Kaplan-Meier analysis was performed for bFFS, MFS and OS while GI, GU and erectile dysfunction adverse events were analyzed by SPSS v.24.

## Results

From 2013 to 2018, there were 96 patients treated with primary HIFU at our institution. We identified 12 patients (12.5%) who subsequently underwent salvage EBRT after primary HIFU treatment. Patient characteristics are summarized in **Table 1**. The median age at initial diagnosis was 68 (range 57-75). Prior to HIFU, all patients except one had disease limited to the prostate (stage T1c-T2a), and all patients had a Gleason score of 7. The median PSA at the time of diagnosis was 7.5 ug/L (range 2.9-14.4). Therefore, all patients except one fell into the intermediate risk category at the time of initial diagnosis, pre HIFU. After HIFU, the median PSA dropped to 4.1 ug/L (range 0.9-11.7) and the median time to failure after HIFU was 13.5 months (range 6-42).

**Table 1:** Baseline patient characteristics at diagnosis.

Patient Number	Age at initial diagnosis	T-stage (AJCC 8^th^ edition)	Gleason Score	Initial PSA (ug/L)	PSA post-HIFU (ug/L)	Time to failure post-HIFU (months)
1	63	3a	7(3 + 4)	3.5	1.3	6
2	64	2a	7(3 + 4)	4.7	3.7	14
3	63	1c	7(4 + 3)	11.4	2.3	35
4	66	1c	7(3 + 4)	6.2	3.6	6
5	53	1c	7(4 + 3)	2.9	0.9	42
6	73	1c	7(3 + 4)	4.7	5	7
7	70	2a	7(3 + 4)	14.4	5.35	23
8	74	1c	7(4 + 3)	8.8	3.4	11
9	71	1c	7(3 + 4)	13.4	11.67	12
10	70	1c	7(4 + 3)	6.13	11.4	17
11	75	1c	7(4 + 3)	13	4.42	24
12	57	1c	7(4 + 3)	9.7	5.48	13

At the time of SRT, all patients had documented radiologic failure on conventional and/or functional imaging. Six patients (50%) had functional imaging (5 had PET prostate-specific membrane antigen (PSMA) and 1 had PET choline). In addition, seven patients (58.3%) had stage migration from intermediate to high-risk disease. In addition, three patients (25%) were found to have low-volume metastatic disease, with one patient (8.3%) experiencing a bone metastasis and two (16.7%) experiencing lymph node metastases. Three patients (25%) had received salvage HIFU prior to SRT. The median time from first HIFU to salvage HIFU procedure was 9 months.

SRT was delivered as either conventional (76-78 Gy in 38-39 fractions, n=11) or hypofractionation (66 Gy in 22 fractions, n=1). Two patients (16.7%) received a simultaneous integrated boost to gross positive disease. ADT was also used in the salvage setting in 75% of the population (n=9), however, data regarding specific duration of ADT were not available. The mean PSA nadir post-RT was 1.2 ug/L (0.1-2.6 ug/L). There was one patient who was currently receiving SRT at the time of analysis, therefore 11 patients were included in the PSA post-RT analysis. Data on failure events and salvage treatment received are summarized in **Table 2**.

**Table 2:** Failure events and salvage treatments.

Patient Number	Time to failure post-HIFU* (months)	Failure Event post-HIFU	Salvage RT dose (Gy) and fractionation received	Salvage ADT received	PSA Post-RT (ug/L)
1	6	Residual lesion on MRI with biopsy showing GS 9(4 + 5)	76/38 to prostate and LN	Yes	<0.1
2	14	Seminal vesicle involvement on PET choline, suspicious nodal involvement, PSA 28, biopsy not repeated and original GS 7(3 + 4)	76/38 with 9.2Gy boost to gross disease	Yes	2.5
3	35	Nodule at previous site of HIFU on MRI with new extra-prostatic extension, biopsy not repeated and original GS 7(4 + 3)	76/38 to prostate and LN	No	0.1
4	6	Residual lesion on MRI with persistent positive biopsy without stage migration, GS 7(3 + 4)	66/22 to prostate	No	2.6
5	42	Recurrence in prostate and pelvic lymph node seen on PET PSMA, biopsy showed GS 8(4 + 4)	76/38 with 8.05Gy boost to gross disease	Yes	N/A
6	7	Residual lesion on MRI and PET PSMA with biopsy showing GS 8(4 + 4)	76/38 to prostate and LN	Yes	<0.1
7	23	Recurrence in prostate and pelvic lymph node seen on PET PSMA, PSA 38	76/38 to prostate and LN	Yes	<0.008
8	11	Residual lesion on MRI with persistent positive biopsyWithout stage migration, GS 7(3 + 4)	78/39 to prostate	Yes	<0.01
9	12	Residual lesion on MRI with biopsy showing GS 9(4 + 5)	76/38 to prostate and LN	Yes	<0.008
10	17	Recurrence in prostate with oligometastatic disease: L1 bone metastasis seen on PET PSMA, PSA 57, biopsy not repeated and original GS 7(4 + 3)	76/38 to prostate and LN	Yes	0.1
11	24	Recurrence on MRI and PET PSMA with biopsy showing GS 8(4 + 4)	76/38 to prostate and LN	Yes	0.1
12	13	Residual lesion on MRI with persistent positive biopsy, GS 7(4 + 3)	78/39 to prostate	No	2.05

*Patients 3,5,6 had HIFU twice, therefore time to failure is taken as time after second procedure. N/A, not applicable.

**Figure 1:**
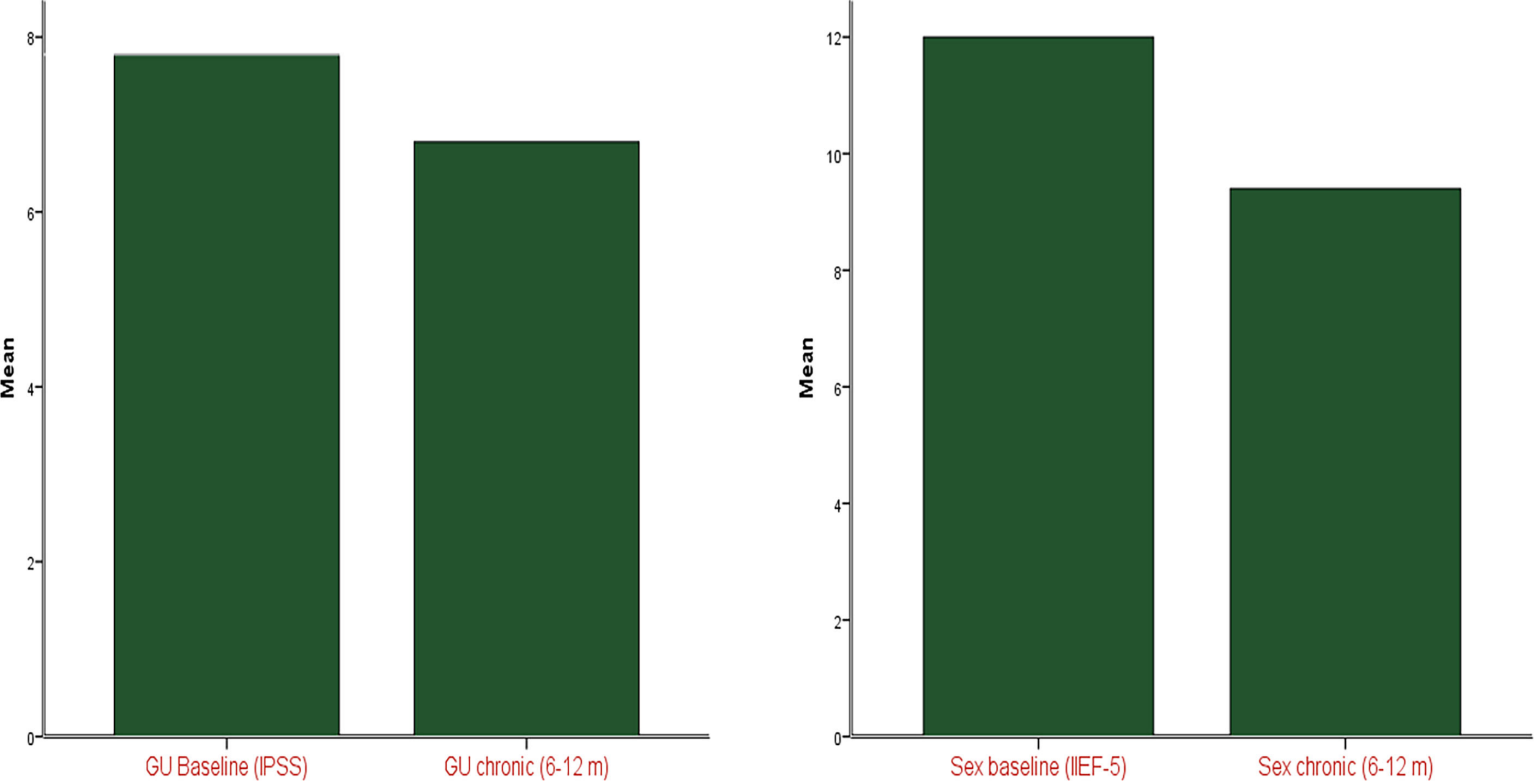
Change in IIEF and IPSS scores from baseline post-RT.

**Figure 2:**
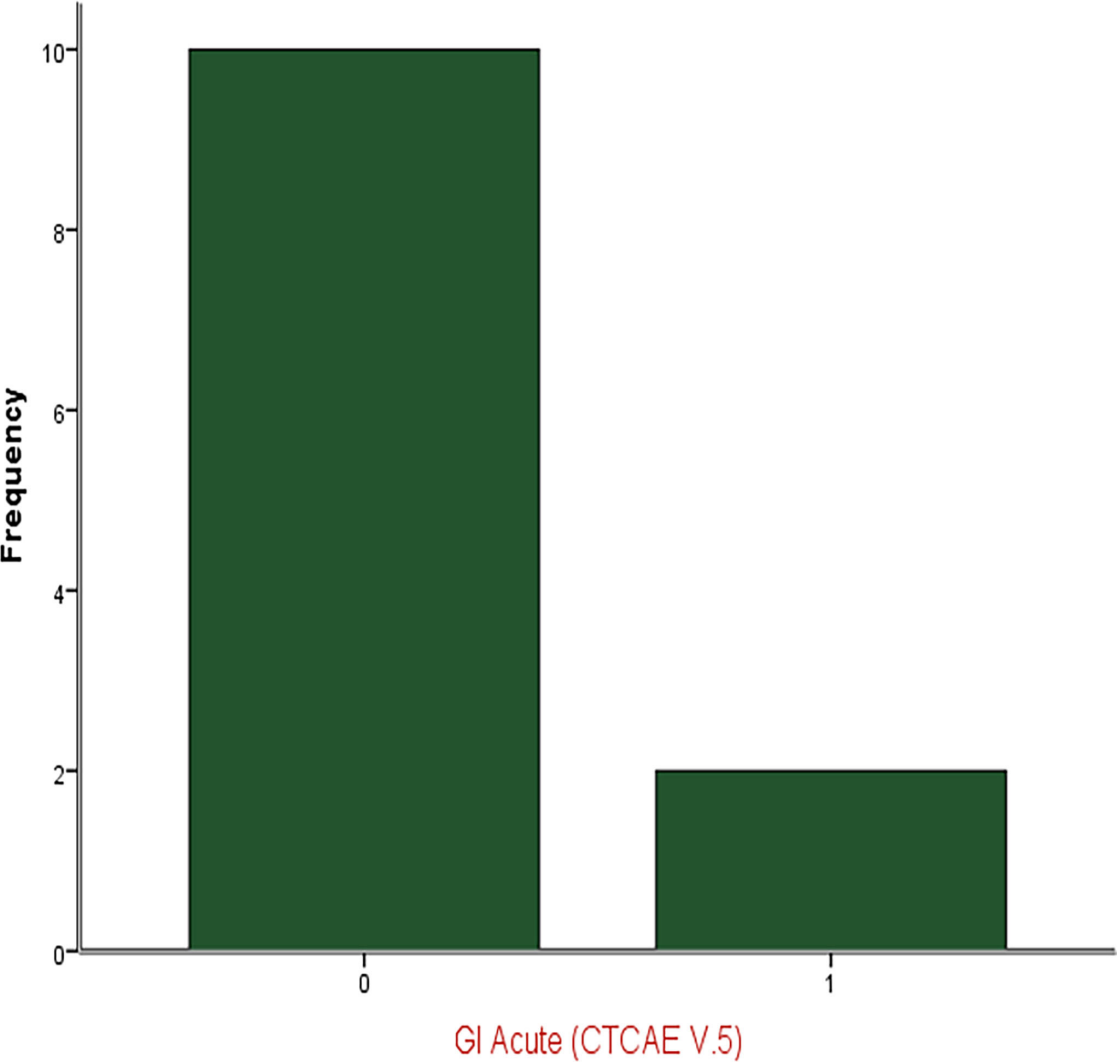
Acute GI Toxicity.

Median baseline IPSS recorded prior to RT was 6 (range 1-23), and median baseline IIEF score was 14 (range 5-24). Post SRT, acute toxicities were all mild, with 5 patients (41.7%) experiencing grade 1 GU toxicity and 2 patients (16.7%) experiencing grade 1 GI toxicity. There were no grade 2 or higher acute toxicities reported. Of the patients who did not have missing data (n=9), there was no reported grade 2 or higher late GI or GU toxicity. For patients with available long-term toxicity data, median IPSS score was 5 (range 2-10) and median IIEF score was 5 (range 5-21). Therefore, IPSS as well as IIEF scores were not significantly different compared to baseline (p = 0.5 and 0.1, respectively), likely related to small patient numbers. Mean IPSS and IIEF scores are shown in **Figure 1**. Acute GI and GU toxicities are reported in **Table 3** and acute GI toxicity is shown in **Figure 2**.

**Table 3:** Acute toxicity after EBRT.

Acute <6 monthsCTCAE v5.0	Grade 0	Grade 1	Grade 2	Grade 3+
Genitourinary	7	5	0	0
Gastrointestinal	10	2	0	0

**Figure 3:**
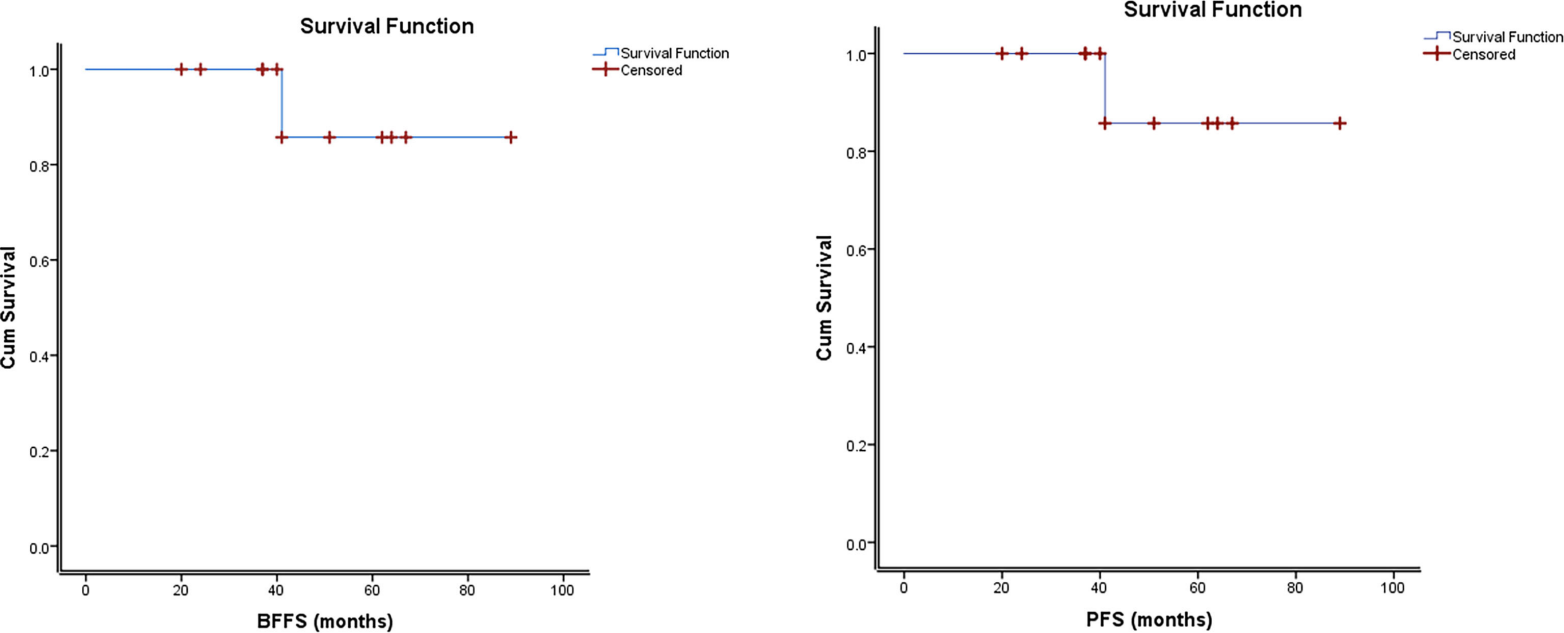
Kaplan Meier Estimates of Biochemical Failure-Free Survival and Progression–Free Survival.

The median follow-up after RT was 46 months. There was one patient who had not yet completed radiotherapy treatments at the time of this analysis, and three others who completed SRT within 6 months of this analysis. Only one patient in this series experienced a progression event, with biochemical and radiological recurrence at 41 months following initial diagnosis. Therefore, the 5-year bFFS and metastasis-free survival were both 83.3%. There were no deaths at the time of this analysis. Kaplan Meier curves are shown in **Figure 3**. 

## Discussion

Due to acceptable GI and GU toxicity, HIFU is sometimes used in select patients who do not undergo primary surgery or RT for localized prostate cancer. However, international guidelines consider the use of HIFU investigational in this setting (1–3). In addition, there is no consensus defining the criteria for failure post-HIFU. Most studies use ASTRO’s Phoenix criteria to define biochemical failure post HIFU. The Stuttgart definition of biochemical failure (PSA nadir + 1.2 ng/mL) is an additional predictor of clinical failure that can be used in the post-HIFU setting (15, 20). Few consensus recommendations include biopsies, PSA and multiparametric MRI for post-HIFU failure detection (21, 22). For patients who recur locally after HIFU, salvage can be achieved with either surgery or RT, with specific consideration required for long-term toxicity (13–18).

From 2013 to 2018, 12 patients who had evidence of local recurrence after primary HIFU received SRT with modern highly conformal EBRT in the form of IG-IMRT. At a median follow-up of 46 months, this technique led to decent oncologic outcomes with tolerable toxicity. To our knowledge, this is one of the largest series reporting on modern highly conformal SRT post HIFU failure. Other comparable published series investigating salvage EBRT after primary HIFU failure are summarized in **Table 4** (14, 15, 19, 23–25).

**Table 4:** Studies exploring salvage RT outcomes after HIFU failure.

Study (Year)	Number of patients included in final analysis	RT median dose (Gy) and technique	Interval between HIFU and RT (months)	Median follow-up (months)	Oncologic outcome	Acute GU Toxicity	Acute GI Toxicity
Pasticier et al. (2008) (19)	32	71 Conformal	12 (mean)	40	5-year DFS 64%	G1 60% G2 8.9% G3+ 2.2%	G1 46.7% G2 13.3% G3+ 0%
Riviere et al. (2010) (23)	100	72 Conformal	10 (median)	33	5-year PFS 72.5%	G1 22% G2 33.7% G3+ 3%	G1 24% G2 15% G3+ 0%
Ripert et al. (2012) (14)	6	74 Conformal	11.7 (mean)	36.5	3-year DFS 83.3%	–	–
Munoz et al. (2013) (24)	24	76 Conformal	–	40.3	3-year bDFS 77.8%	G1 33.3% G2 12.5% G3+ 12.5%	G1 25% G2 1% G3+ 0%
Alongi et al. (2014) (25)	15	71.4 IMRT	30 (median)	12	1-year bRFS 80%	G1 47% G2 27% G3+ 0%	G1 20% G2 13% G3 0%
Rigo et al. (2020) (15)	MHRT 16 SBRT 8	MHRT 71.4 SBRT 32.5 IMRT	39 (median)	28	3-year bRFS 70%	G1 50% G2 0% G3 0%	G1 0% G2 12.5% G3 0%

Non-modern radiotherapy techniques were used in early published data reporting on salvage radiotherapy after HIFU failure. Pasticier et al. reviewed 45 patients treated with SRT from 1995 to 2004 with a mean interval to failure after HIFU of 12 months (19). In this cohort, salvage treatment consisted of 5-field conformal SRT (with or without ADT) delivering a median dose of 71Gy in conventional fractionation to the prostate. At a median follow-up of 40 months, the 5-year disease free survival (DFS) was 64%. Similarly, Riviere et al. reviewed 100 patients who underwent SRT after HIFU from 1995 to 2008 (23). Patients were treated using conformal SRT with a median dose of 72Gy. The 5-year PFS was 72.5% at a median follow-up of 33 months. In both studies, acute GI and GU toxicities were mild, with the majority of patients experiencing grade 2 or lower toxicity (19, 23). Ripert et al. reported on seven patients that received conformal SRT at a dose of 74Gy after HIFU failure at a single institution from 2004 to 2008 (14). At a median follow-up of 36.5 months, and after exclusion of one patient who died of unrelated causes, the 3-year DFS was 83.3%. Acute toxicities were not reported, but chronic toxicities remained mild (14). Munoz et al. also reviewed a series of 24 patients who underwent conformal SRT after HIFU failure and demonstrated biochemical disease-free survival (bDFS) at 3 years of 77.8% (24).

To our knowledge, only 2 other published studies have used IMRT in the salvage setting after HIFU. Alongi et al. evaluated 15 patients who had 11-choline PET detected intraprostatic-only failure after HIFU (25). Patients received moderately hypofractionated RT (MHRT) delivered in 28 fractions *via* inverse planned IMRT. At a median follow-up of 12 months, 3 out of 15 patients had nodal recurrence. Acute GU and GI toxicities after IMRT were similar to those reported in previous studies using conformal techniques. More recently, Rigo et al. reviewed 24 patients that experienced localized failure after HIFU (15). Salvage RT was either delivered as MHRT (n=16) or as extremely hypofractionated stereotactic body RT (SBRT, n=8). Acute toxicity profiles were favorable with both approaches, with GU toxicity limited to grade 1, and only 3 patients experiencing radiation-induced proctitis. The biochemical relapse-free survival (bRFS) was 70% at 3 years (15).

In our study, the 5-year bFFS and MFS were both 83.3%, and there were no deaths at the time of this analysis. At a median follow-up of 46 months, our follow-up time is the longest among other published studies. Despite our heterogeneous, mostly high-risk patient population, our reported oncologic outcomes are similar to other published studies.

Acute toxicities reported in our patients remained acceptable, with none reporting grade 2 or higher toxicities. In fact, the majority of patients did not report acute GI toxicity, while 5 patients (41.7%) reported grade 1 acute GU toxicity. This is markedly lower than those reported in other studies where conformal techniques were used, but similar to rates reported by Rigo et al. (15). For patients with available long-term toxicity data, median IPSS score was similar to baseline. Although not statistically different, baseline IIEF scores were higher than those reported after SRT. This decline in sexual function is likely multifactorial and possibly related to increasing age and concomitant ADT use. While we cannot directly compare with other studies mentioned above, studies comparing IMRT versus conformal techniques have shown a reduction in both acute and late GI and GU toxicity in patients undergoing IMRT for localized prostate cancer (26, 27). Zelefsky et al. showed that IMRT reduced the 10-year risk of GI toxicity from 13% to 5% compared to conformal RT. In addition, acute GU toxicity was a predictor of development of late GU toxicity (27).

While this study yields important insight into the management and outcomes of local failure post-HIFU, it has notable limitations. Firstly, inherent to its retrospective nature, there were important data missing in certain circumstances. For example, there were 4 patients that completed RT within 6 months at the time of this analysis, therefore making it difficult to assess oncologic outcomes in these patients. Given the retrospective nature of this study, we could not draw conclusions on patients that were lost to follow-up shortly after EBRT, and these patients were ultimately excluded from our analysis, limiting the interpretation of our results. Reporting on late toxicities was also limited by missing data, for example total duration of ADT. Given that this is single institutional data, the total number of patients included in our analysis is small. However, to our knowledge, this is still one of the largest series reporting on modern SRT post HIFU failure. We used advanced techniques of highly conformal dose escalated RT with reported toxicity data and modest follow-up time. Despite these limitations, our oncologic outcome data remain favorable, with a bFFS and metastasis-free survival rate of 83.3%.

While our study is limited by a small number and relatively short follow up, our review endorses salvage EBRT as an effective and safe treatment option in localized prostate cancer patients failing primary HIFU. Given the lack of large prospective studies in this setting, our results suggest that salvage EBRT may be safely offered to these patients. Further studies assessing and comparing the oncological outcomes of salvage EBRT and radical prostatectomy are needed. Furthermore, our study focused on specific outcomes of salvage EBRT after HIFU failure without addressing the actual failure rates of HIFU, which is not uncommon. Therefore, future studies are needed to better understand the incidence, location and cause of HIFU failure.

## Data availability statement

The raw data supporting the conclusions of this article will be made available by the authors, without undue reservation.

## Author contributions

All authors contributed to the study conception and design. Material preparation, data collection and analysis were performed by VL, SE and MT. The first draft of the manuscript was written by VL and all authors commented on previous versions of the manuscript. All authors contributed to the article and approved the submitted version.

## Conflict of interest

The authors declare that the research was conducted in the absence of any commercial or financial relationships that could be construed as a potential conflict of interest.

## Publisher’s note

All claims expressed in this article are solely those of the authors and do not necessarily represent those of their affiliated organizations, or those of the publisher, the editors and the reviewers. Any product that may be evaluated in this article, or claim that may be made by its manufacturer, is not guaranteed or endorsed by the publisher.
